# Neurotensin Receptor-1 Expression in Human Prostate Cancer: A Pilot Study on Primary Tumors and Lymph Node Metastases

**DOI:** 10.3390/ijms20071721

**Published:** 2019-04-07

**Authors:** Clément Morgat, Adrien Chastel, Vincent Molinie, Romain Schollhammer, Gaétan Macgrogan, Valérie Vélasco, Bernard Malavaud, Philippe Fernandez, Elif Hindié

**Affiliations:** 1 CNRS, INCIA, UMR 5287, F-33000 Bordeaux, France; adrien.chastel@chu-bordeaux.fr (A.C.); romain.scho@gmail.com (R.S.); philippe.fernandez@chu-bordeaux.fr (P.F.); elif.hindie@chu-bordeaux.fr (E.H.); 2 University of Bordeaux, INCIA, UMR 5287, F-33000 Bordeaux, France; 3 Department of Nuclear Medicine, University Hospital of Bordeaux, F-33000 Bordeaux, France; 4 Department of Pathology, University Hospital of Martinique, 97261 Fort de France, France; Vincent.MOLINIE@chu-martinique.fr; 5 Surgical Pathology unit, Department of BioPathology, Institut Bergonié, F-33076 Bordeaux, France; G.MacGrogan@bordeaux.unicancer.fr (G.M.); V.Velasco@bordeaux.unicancer.fr (V.V.); 6 INSERM, ACTION U1218, F-33076 Bordeaux, France; 7 Department of Urology, University Hospital of Toulouse, F-31000 Toulouse, France; bernard.malavaud@me.com

**Keywords:** neurotensin receptor-1, NTR_1_, prostate cancer, neuropeptide

## Abstract

Neurotensin and its high-affinity receptor, NTR_1_, are involved in the growth of various tumors. Few data are available regarding NTR_1_ expression in normal and tumoral human prostate tissue samples. NTR_1_ expression was assessed using immunohistochemistry in 12 normal prostate tissues, 11 benign prostatic hyperplasia (BPH), 44 prostate cancers, and 15 related metastatic lymph nodes (one per patient, when available). NTR_1_-staining was negative in normal prostate and BPH samples. NTR_1_ was overexpressed in four out of 44 (9.1%) primary tumors. There was no clear association between NTR_1_ overexpression and age, PSA-values, Gleason score, pT-status, nodal-status, or margin. NTR_1_ was expressed at a high level of five out of 15 (33.3%) metastatic lymph nodes. NTR_1_ overexpression was thus more frequent in metastatic lymph nodes than in primary tumors (*p* = 0.038). In this limited series of samples, NTR_1_ overexpression was observed in few primary prostate cancers. Upregulation was more frequent in related lymph nodes. The presence of this target in metastatic lymph nodes may open new perspectives for imaging and radionuclide therapy of prostate cancer. Factors driving NTR_1_ expression in primary prostate cancer and in nodal and distant metastases still need to be characterized.

## 1. Introduction

Prostate cancer is the most frequent cancer in men. It is initially an androgen-dependent disease that may progress to an androgen-independent stage: castration resistant prostate cancer (CRPC) with poor prognosis related to the reactivation of androgen-receptor (AR) transcriptional activity [1,2]. Drugs targeting androgen and their receptors in CRPC have been approved but resistance limits their successes. Alternative pathways for the proliferation of prostate cancer cells have been identified, such as the neurotensin/neurotensin receptors axis [3].

Neurotensin effects are mediated through three receptor subtypes: NTR_1_ (high-affinity receptor) and NTR_2_ (low-affinity receptor), both G-protein coupled receptors, and NTR_3_, a single transmembrane domain. Physiologically, neurotensin is released from endocrine cells (N cells) in response to lipid ingestion and is involved in the stimulation of pancreatic, biliary, and gastric acid secretions; the facilitation of fatty acid absorption; and the regulation of small-bowel motility [4]. Human prostate cancer cells exhibit a growth response to subnanomolar concentrations of neurotensin [3]. After binding to its high-affinity receptor, NTR_1_, neurotensin stimulates mitosis of cancerous prostatic cells through Src-, MMP-, and PKC-dependent ligand-mediated transactivation of EGFR, which mediate IGF-1R phosphorylation and stimulation of the MAP kinase pathway in a PI3K-dependent manner [5,6,7]. Moreover, the selective NTR_1_ antagonist SR48692 is able to inhibit the neurotensin-induced growth of prostate cancer cells [6], suggesting the involvement of NTR_1_ as an oncogenic receptor in prostate cancer. Neurotensin has been showed to mediate neuroendocrine differentiation of prostate cancer [8]. Receptor subtypes involved in this mechanism are still a matter of debate [8,9]. Interestingly, androgen deprivation induces acute neurotensin production, suggesting the involvement of neurotensin and its receptors in androgen-independent prostate cancer and/or neuroendocrine differentiation of prostate cancer [8,9,10]. Non-tumoral cells in the microenvironment producing neurotensin are in the spotlight [8]. To assess the potential applications of neurotensin receptors, NTR_1_ expression has been analyzed in several prostate cancer cell models with controversial results. NTR_1_ expression has been highlighted in both malignant and non-malignant cells with the highest expression in malignant cancerous cells [11]. In this study, the authors also demonstrated that malignant cells with a basal phenotype express a higher level of NTR_1_, and in a benign tissue section, that NTR_1_ was stained in basal and luminal compartments as well as in stroma [11]. Finally, the authors conclude that NTR_1_ expression is modulated by the overall differentiation state, which is not directly influenced by androgens [11]. However, expression of NTR_1_ at the mRNA level does not necessarily translate into similar results at the protein levels [12,13].

In light of these conflicting results and given the paucity of data on human prostate cancer samples, we investigated in this pilot study NTR_1_ expression in samples of normal human prostate tissues, benign prostatic hyperplasia (BPH), primary prostate cancer, and metastatic lymph nodes.

## 2. Results

**Figure 1:**
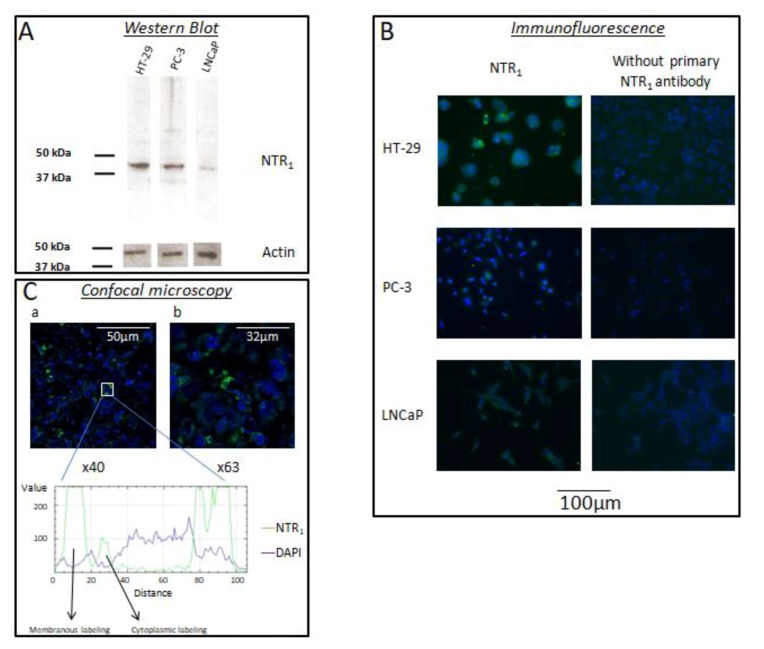
Expression profile of NTR_1_ in prostate cancer cell lines PC-3 and LNCaP and the colonic cell line HT-29 (positive control). (**A**) Western blot of NTR_1_ showing high expression in HT-29 and PC-3 cells and weak expression in LNCaP cells. (**B**) NTR_1_ immunofluorescence revealing cytoplasmic and membranous labelling in HT-29 and PC-3 cells. (**C**) Confocal microscopy (40× and 63× magnifications) of a PC-3 xenograft confirming cytoplasmic and membranous labelling.

Analysis of Western blots revealed that a single band is visible at the expected NTR_1_ molecular weight on PC-3 and HT-29 cell lines. LNCaP cells weakly express NTR_1_ (Figure 1A). Subcellular localization of NTR_1_ was assessed by immunocytofluorescence. Staining was mainly cytoplasmic and granular, often accompanied with a membranous labelling in positive cell lines (Figure 1B). Immunocytochemistry results revealed that formalin-fixed PC-3 cells express NTR_1_, whereas formalin-fixed LNCaP cells exhibit weak NTR_1_ expression. Finally, to assess whether the tumoral environment may modify NTR_1_ expression, we looked at its expression in a PC-3 xenograft using confocal microscopy. NTR_1_ expression seems to be higher in xenograft than in cultured cells. Staining was cytoplasmic and granular, often accompanied with a membranous labelling. Figure 1C shows confocal microscopy of the PC-3 xenograft. Study of the NTR_1_ signal across a single cell confirmed that NTR_1_ expression is cytoplasmic and accompanied by a membranous labelling.

**Figure 2:**
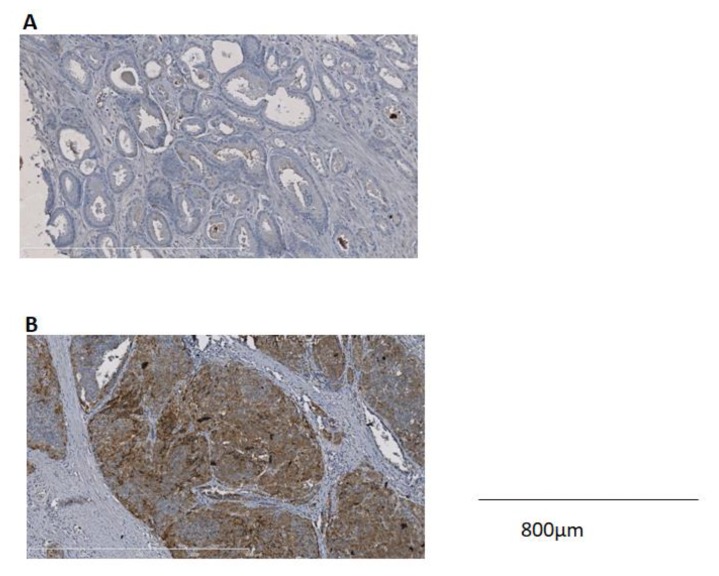
Expression profiles of NTR_1_ in prostate cancer. (**A**) No NTR_1_ expression in Gleason 6 prostate cancer. (**B**) Strong NTR_1_ staining in Gleason 8 prostate cancer. Images were obtained with 10× magnification.

Normal prostate samples and BPH samples either did not express or showed weak expression of NTR_1_. Neural cells are often stained for NTR_1._ As regards primary tumors of prostate cancer cases, NTR_1_ overexpression (≥10% of stained tumor cells) was seen in 4 of 44 cases (9.1%). Among the other cases, NTR_1_ staining was either absent (30 cases) or weak (<10% of tumor cells stained). NTR_1_ staining was granular and mainly cytoplasmic (Figure 2).

Stromal cells did not express NTR_1_. The tumor presenting the highest NTR_1_ expression was a poorly differentiated prostatic adenocarcinoma (CK20−, CK7−, PSAp+, p63−, p504s+) without neuroendocrine differentiation (CgA-, synaptophysin-), Gleason 9, node-positive, T_3_, R_0_. This patient was 63 years old with a PSA value below 10 ng/mL.

Table 1 shows that none of the studied parameters (age, PSA-values, pT-status, N-status, Gleason score, margin status) were associated significantly with NTR_1_-expression.

The rate of NTR_1_-overexpression in metastatic lymph nodes (33.3%) was higher than in primary tumors (9.1%) (*p* = 0.038) (Table 2).

**Figure 3:**
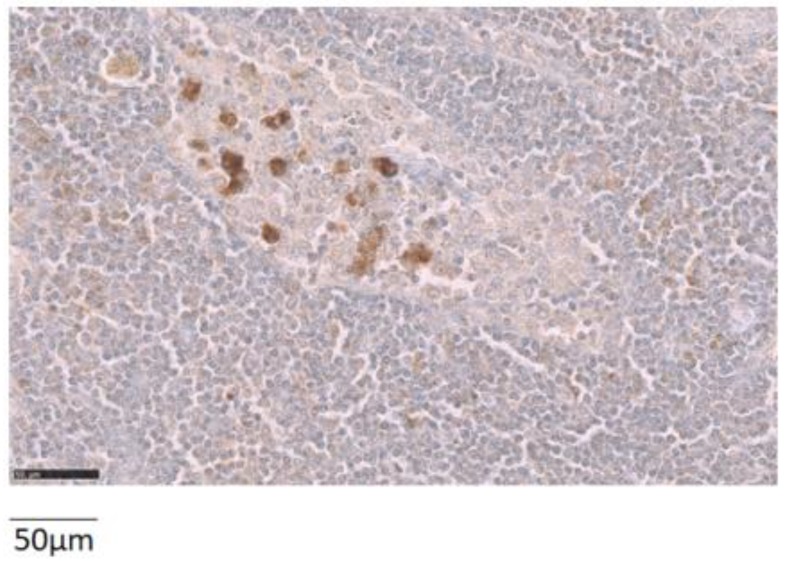
Representative example of the NTR_1_ expression in a metastatic lymph node. The image was obtained with 40× magnification.

A lymph node metastasis was available for analysis in 15 patients. NTR_1_ was overexpressed in five of the 15 cases (33.3%) (Figure 3).

In two out of these five cases, both the primary tumor and nodal metastasis overexpressed NTR_1_, while in three cases only the lymph node metastasis overexpressed NTR_1_. In addition, there was only one case where the primary tumor expressed NTR_1_ while the lymph node did not.

## 3. Discussion

Data regarding NTR_1_ expression in prostate cancer are limited mainly to studies on cell lines [11,12,13] with conflicting results. Herein, we confirm the upregulation of NTR_1_ in the PC-3 model compared to LNCaP cells [10,14]. Whether the absence of AR and/or prostate-specific membrane antigen (PSMA) and/or the differentiation state in PC-3 cells is linked to the overexpression of NTR_1_ remains to be explored. The NTR_1_ receptor is mainly cytoplasmic in prostate cancer cells as shown by the granular labelling, as also described in some other malignancies [15,16]. This immunostaining pattern may be judged as non-classical by some experts [17], but an original mechanism by which NTR_2_ is able to promote cytoplasmic retention of NTR_1_ has been described [18]. In our hands, PC-3 cells also express the NTR_2_ subtype (on Western blot, data not shown) as already reported [19]. Moreover, a chronic exposure to the peptide neurotensin is able to activate the NTR_1_ gene [15]. Under these conditions, NTR_1_ is accumulated in the perinuclear recycling compartment (cytoplasmic staining) and then addressed to the cell membrane [20,21]. Our granular and cytoplasmic staining of NTR_1_ in prostate cancer cells is in agreement with the proposed mechanisms.

Then, we assessed the expression of the NTR_1_ protein by immunohistochemistry in a pilot series of primary prostate cancer cases and lymph nodes metastases if present. Our data show that about 10% of primary prostate cancers overexpressed NTR_1_. NTR_1_ expression was not associated with any clinical, biological, or pathological parameters, but the number of samples is quite small. There was a trend for a higher rate of NTR_1_ expression in patients with a higher Gleason score or from primary prostate cancer from node-positive patients. Preliminary data in the literature incriminate neurotensin and its receptors in prostate cancer with neuroendocrine differentiation [8,10]. In this study, the tumor with the highest NTR_1_ expression did not exhibit a neuroendocrine profile. A specific study of NTR_1_ expression in neuroendocrine prostate cancer would be of interest as these tumors may have lost PSMA expression. Even so, such cases account only for a small number of prostate cancers [22]. Loss of PSMA with ^18^F-FDG-avidity at discordant sites has also been reported in PSMA-targeted radionuclide therapy studies [23]. Moreover, investigation of molecular cross-talk between hormone receptors and/or PSMA and NTR_1_ would be helpful to better understand the regulation of NTR_1_ expression in prostate cancer.

Most importantly, the therapy potential offered by targeting NTR_1_ relies on a better understanding of the NTR_1_ expression in regional and distant metastases. We were able to study lymph node metastases from 15 patients (one lymph node per case). The rate of NTR_1_ overexpression is higher in these metastatic tissues (33.3%) than in the overall series of primary tumors (*P* = 0.038). Moreover, the number of tumoral cells expressing NTR_1_ in metastatic lymph nodes is also higher than in primary tumors (66.0 ± 31.3% versus 12.5 ± 5%, respectively). Factors driving upregulation of NTR_1_ in metastatic tissues remain to be elucidated.

NTR_1_ has been found to be expressed in metastases of other cancers, such as pancreatic cancer and hepatocellular carcinoma [24,25]. It has been suggested that co-expression of NTR_1_ and its endogenous peptide constitutes an important stimulus promoting cancer invasion and metastases [25,26]. There might also be an implication in prostate cancer. Neurotensin is indeed able to stimulate prostate cancer cell growth, while the selective neurotensin antagonist (SR48692) decreases prostate cancer cell growth [6]. Therefore, pharmacological blockade of NTR_1_ using antagonists might prove to be useful. It is now necessary to elucidate the place of NTR_1_ targeting in the current prostate cancer management strategy, notably in high-risk prostate cancer patients. Several radiolabelled probes are under investigation in this setting, such as small molecules targeting PSMA [27]. For future targeted therapy, NTR_1_ expression should also be assessed on samples of distant metastases from untreated and castration-resistant prostate cancer patients. Although the frequency of NTR_1_ expression in primary tumors and metastatic lymph nodes appears to be lower than that of PSMA, investigating PSMA-negative (on IHC or PET imaging) prostate tumors would be of particular interest. Therefore, imaging and therapy of prostate cancer patients with the recently developed radiolabelled NTR_1_ analogues [28,29,30] can offer a complete insight on the role of this approach.

The main limitation of our study is the low number of primary tumors and metastatic lymph nodes studied and validation of these data on a larger series is needed. The prognostic value of NTR_1_ in prostate cancer still needs to be addressed.

## 4. Materials and Methods

### 4.1. Antibody Used

The anti-NTR_1_ antibody used was a goat polyclonal antibody directed against the human COOH terminus of the receptor (C-20; Santa Cruz Biotechnology, Inc., Heldeiberg, Germany), as used by others [15]. Rules for G-protein coupled receptor immunohistochemical (IHC) studies in human tissues have been established previously [17]. Our methodology was as follows: (i) Western blotting of prostate cancer cell line lysate for antibody specificity, and (ii) immunochemistry of the cell line fixed and embedded with the same material used for the human cancer sample to evaluate the influence of the fixative, subcellular localization of the immunostaining. Finally, our series of human normal and cancerous prostate samples was analyzed regarding NTR_1_ expression.

### 4.2. Cell Lines

For all experiments, the human colonic cell line HT-29 was used as a positive control. The human prostate cancer cells LNCaP (lymph node from metastatic prostate adenocarcinoma, AR^+^, PSMA^+^) and PC-3 (bone metastasis from high grade prostate cancer; AR^−^, PSMA^−^) have been used in this study to explore NTR_1_ expression in prostate cancer. PC-3 cells were cultured at 37 °C and 5% CO_2_ in DMEM/F12 (Gibco, Illkirch, France) medium supplemented with 10% (*v*/*v*) FBS, 100 U/mL penicillin, and 100 µg/mL streptomycin. LNCaP and HT-29 cells were cultured at 37 °C and 5% CO_2_ in RPMI 1640 (Gibco) medium supplemented with 10% (*v*/*v*) FBS, 100 U/mL penicillin, and 100 µg/mL streptomycin. Cells were then submitted to Western blotting, immunofluorescence, immunochemistry, and xenografting in nude mice (PC-3 cells).

### 4.3. Xenograft

All animal experiments/protocols were performed in accordance with the guidelines of the Institute of Nuclear Medicine and Allied Sciences (INMAS) animal ethics committee (Regn. no: 8/GO/a/99/CPCSEA, approval date: 13 November 2011). The animals were euthanized using cervical dislocation and all animal experiments were performed in accordance with the relevant laws (Animals ACT 1986) and the guidelines of the INMAS animal ethics committee (no. INM/DASQA/1AEC/09/15, approval date: 9 September 2015). The institutional animal ethics committee approved the experiments. 5 × 10^6^–6 × 10^6^ of PC-3 cells (100 µL) in 50% Matrigel in PBS *v*/*v* were injected to the left flank of nude mice. Tumors were obtained within a period of three to four weeks. The PC-3 tumor was excised and was formalin-fixed and paraffin-embedded for confocal microscopy. Distribution of the NTR_1_ signal across a single cell was analyzed using Image-J (version 1.50i, NIH, Bethesda, MD, USA).

### 4.4. Western Blotting

NTR_1_ expression was investigated by Western blotting. Cell lines were lysed using RIPA buffer. Membrane proteins were denatured by boiling at 85 °C for 5 min using 4× Laemmli sample buffer. Protein samples were submitted to electrophoresis for 60 min at a constant 200 V on SDS polyacrylamid gel and subsequently electroblotted onto PVDF membrane for 90 min at a constant 300 mA current. The membranes were blocked in PBS containing 5% (*w*/*v*) BSA for 1 h at room temperature and were incubated overnight at 4 °C with rabbit polyclonal anti human NTR_1_. The signal was finally recorded using autoradiography films.

### 4.5. Immunofluorescence

HT-29, LNCaP, and PC-3 cells were grown for two days at 37 °C and 5% CO_2_, respectively. Non-specific binding sites were blocked in PBS containing 1% BSA and 0.3% Triton X-100 for 90 min. After washing, cells were incubated with the primary antibody anti-human NTR_1_ (1/500) at room temperature overnight and then with FITC-labelled donkey anti-goat secondary antibody for 90 min. Nuclei were labelled with DAPI. For each experiment, non-specific binding was evaluated by omitting the primary antibody. The cells were imaged using a Leica immunofluorescence microscope and an Olympus camera at 20× magnification.

### 4.6. Immunochemistry Procedure

NTR_1_-immunochemistry was carried out as described by Souaze and colleagues [15].

### 4.7. Human Samples

Forty-four primary prostate cancer samples and fifteen related metastatic lymph nodes were obtained from the tumor bank of the University Hospital of Toulouse. Twelve normal prostate samples and 11 benign prostatic hyperplasia samples from other patients were also examined for comparison. Patient samples were obtained after informed consent in accordance with the Declaration of Helsinki and stored at the “CRB Cancer des Hôpitaux de Toulouse (BB-0033-00014)” collection. According to the French law, CRB Cancer collection has been declared to the Ministry of Higher Education and Research (DC-2008-463) and obtained a transfer agreement (AC-2008-820) after approbation by ethical committees under N°CEBH 2012/40 (approval date: 7 June 2012). Clinical and biological annotations of the samples have been declared to the CNIL (Comité National Informatique et Libertés).

Regarding the 44 cases of prostate cancer, no patient had received hormonal treatment, chemotherapy or radiotherapy prior to surgery. Twenty-five patients were aged 64 years and older; 22 patients had prostate-specific antigen (PSA) serum level ≥10 ng/mL; the Gleason scores were 5 or 6 (3 + 3) in 12 cases, 7 (3 + 4) in six cases, 7 (4 + 3) in six cases, and 8 or 9 in 20 cases; 15 cases were pT_2_, 28 pT_3_, and one pT_4_; 27 were pN_0_ or pN_x_; and 17 were pN^+^. Among the 17 patients with lymph node metastases, a lymph node sample was available for analysis in 15 cases (one node per patient) and constitutes the metastatic lymph node group (two cases were discarded for analysis due to the lack of biological material). NTR_1_ expression was assessed under a light microscope and was scored according to published procedures [15]. The chosen cut-off value of 10% or more of stained cells as a positivity threshold by Souazé et al. has demonstrated clinical value for another internalized target, Prostate Specific Membrane Antigen (PSMA) [31].

### 4.8. Statistical Analysis

In order to study associations between NTR_1_ expression and other parameters, NTR_1_ data were dichotomized into two groups based on the positivity cut-off reported in literature [15]. We studied associations between NTR_1_ expression and various clinical, pathological, and biological parameters in cases with prostate cancer (age, serum PSA level, pT status, lymph node status, Gleason score...). Differences between categorized variables were assessed with the χ^2^ test or Fischer’s exact test as appropriate. All *p*-values are two-sides. *p* < 0.05 was considered statistically significant. All data were analyzed using GraphPad Prism v6.01 (GraphPad software, San Diego, CA, USA).

## 5. Conclusions

In this pilot study, NTR_1_ was overexpressed in a small percentage of primary prostate cancer, and significantly more often overexpressed in metastatic lymph nodes. Factors driving overexpression of NTR_1_ in prostate cancers remain to be discovered. The presence of this target in metastatic lymph nodes may open new perspectives for imaging and radionuclide therapy of prostate cancer that deserve further investigation.

## Figures and Tables

**Table 1:** Association between NTR_1_ expression in primary tumors and clinical and pathological data in 44 patients with prostate cancer.

Variable	NTR_1_ Overexpression	*p*
Age		0.622
≤64 years (*n* = 19)	1/19	
>64 years (*n* = 25)	3/25	
PSA value		1.000
<10 ng/mL (*n* = 22)	2/22	
≥10 ng/mL (*n* = 22)	2/22	
pT-status		1.000
pT2 (*n* = 15)	1/15	
pT3–T4 (*n* = 29)	3/29	
N-status		0.282
N_0–X_ (*n* = 27)	1/27	
N^+^ (*n* = 17)	3/17	
Gleason score		0.358
5–6 (*n* = 12)	1/12	
7 (*n* = 12)	0/12	
8–9 (*n* = 20)	3/20	
Gleason score grouped		0.316
5–7 (*n* = 24)	1/24	
8–9 (*n* = 20)	3/20	
Margin status		0.558
R_0_ (*n* = 33)	4/33	
R_1_ (*n* = 11)	0/11	

**Table 2:** NTR_1_ expression according to the origin of tumor sample.

	NTR_1_ Overexpression	*p*
Tumor sample		0.038
Primary tumor (*n* = 44)	4/44	
Metastatic lymph nodes (*n* = 15 ^†^)	5/15	

^†^ Two samples were not included in the analysis due to the lack of available biological material from two of the 17 node-positive patients.

## Abbreviations

PSMA	Prostate Specific Membrane Antigen
NTR1	Neurotensin receptor-1
NTR2	Neurotensin receptor-2
NTR3	Neurotensin receptor-3
FITC	Fluorescein isothiocyanate
DAPI	4′,6-Diamidino-2-phenylindol
IHC	Immunohistochemistry
PET	Positron Emission Tomography
CK	Cytokeratine
BPH	Benign Prostatic Hyperplasia
CRPC	Castration Resistant Prostate Cancer
